# Prone Position after Liberation from Prolonged Mechanical Ventilation in COVID-19 Respiratory Failure

**DOI:** 10.1155/2020/6688120

**Published:** 2020-11-12

**Authors:** Andrei Karpov, Anish R. Mitra, Sarah Crowe, Gregory Haljan

**Affiliations:** ^1^Division of Critical Care Medicine, Department of Medicine, University of British Columbia, Vancouver, BC, Canada; ^2^Department of Emergency Medicine, University of British Columbia, Vancouver, BC, Canada; ^3^Division of Critical Care Medicine, Department of Medicine, Surrey Memorial Hospital, Surrey, BC, Canada; ^4^Division of Critical Care Medicine, Department of Nurse Practitioners, Surrey Memorial Hospital, Surrey, BC, Canada

## Abstract

**Design:**

This is a retrospective case series describing the feasibility and tolerability of postextubation prone positioning (PEPP) and its impact on physiologic parameters in a tertiary intensive care unit during the COVID-19 pandemic. *Setting and Patients*. This study was conducted on patients with COVID-19 respiratory failure hospitalized in a tertiary Intensive Care Unit at Surrey Memorial Hospital during the COVID-19 pandemic. *Measurements and Results*. We did not find prior reports of PEPP following prolonged intubation in the literature. Four patients underwent a total of 13 PEPP sessions following liberation from prolonged mechanical ventilation. Each patient underwent a median of 3 prone sessions (IQR: 2, 4.25) lasting a median of 1.5 hours (IQR: 1.2, 2.1). PEPP sessions were associated with a reduction in median oxygen requirements, patient respiratory rate, and reintubation rate. The sessions were well tolerated by patients, nursing, and the allied health team.

**Conclusions:**

The novel practice of PEPP after liberation from prolonged mechanical ventilation in patients with COVID-19 respiratory failure is feasible and well tolerated, and may be associated with favourable clinical outcomes including improvement in oxygenation and respiratory rate and a low rate of reintubation. Larger prospective studies of PEPP are warranted.

## 1. Introduction

A recent study by Mitra et al. [1] highlighted a low mortality for patients admitted to the intensive care unit (ICU) with coronavirus disease 19 (COVID-19) respiratory failure. Postextubation prone positioning (PEPP) was used in a subset of patients in that cohort but not previously described.

The rationale for prone positioning of mechanically ventilated patients in ARDS has been previously well described [2–5]. Prone positioning allows for improved oxygenation by recruiting dependent lung regions, thus improving ventilation to perfusion (V/*Q*) matching [6, 7] and reducing fraction of inspired oxygen (FiO_2_) requirements. Prone positioning allows for more optimal redistribution of transpulmonary pressure gradients across the lungs, limiting cyclic atelectasis and ventilator-induced lung injury [7]. Increased recruitment of atelectatic lung may lead to reduced hypoxic vasoconstriction and subsequent improvement in cardiac output [8]. Finally, prone positioning may improve secretion clearance from posterior lung segments in patients who are paralyzed or have impaired cough and airway clearance [5].

Conscious prone positioning garnered renewed interest in the face of the COVID-19 pandemic as a means of improving oxygenation and avoiding intubation [9–12]. Conscious prone positioning has also been described in neonates [13], as well as perioperative patients, as a means of improving secretion clearance and avoiding intubation [14].

Patients admitted to the ICU with COVID-19 respiratory failure often have high illness severity scores and require prolonged mechanical ventilation and concomitant use of steroids and paralytics [1, 15]. These known risk factors for respiratory muscle weakness result in impaired secretion clearance and are associated with increased reintubation rates, mortality, and prolonged ICU length of stay (LOS) [16, 17].

PEPP may be particularly beneficial in this population. Recruitment of previously collapsed lung units may allow for ventral secretion movement into larger airways, facilitating their clearance [18]. Prone positioning also increases chest wall elastance [3], could theoretically improve chest wall mechanics, and augment active expiration during coughing. To our knowledge, PEPP following prolonged mechanical ventilation has not been previously described although it has physiologic rationale.

As evidence used in the management of ICU patients with COVID-19 is rapidly evolving and physicians employ novel treatment strategies to best care for patients, it is critical that the safety and efficacy of different interventions be evaluated. Therefore, the objectives of our study were twofold. First, we aimed to conduct a systematic search of the literature for reports of PEPP following prolonged mechanical ventilation. Our second objective was to evaluate and describe our experience of PEPP in patients affected by COVID-19 as a novel application of a well-tested intervention.

## 2. Methods

The Fraser Health Research Ethics Board approved the protocol for this retrospective case series. Consent for participation was obtained from the patients or their substitute decision maker.

**Figure 1:**
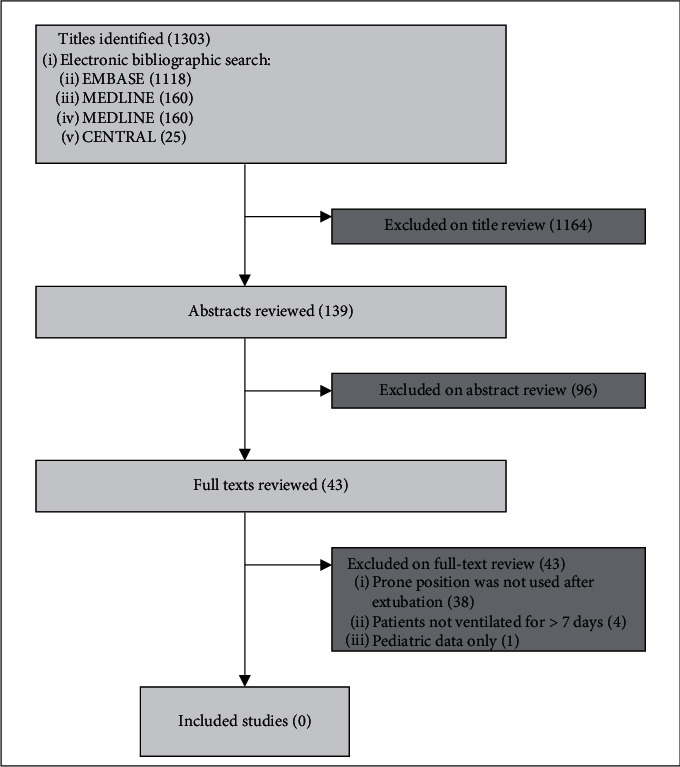
Search flow diagram.

We conducted a literature search in CENTRAL, MEDLINE, and EMBASE through the Ovid interface searching for reports of PEPP following prolonged mechanical ventilation (>7 days). See search diagram and terms in Figure 1 and Appendix.

We reviewed the records of all patients admitted to the ICU at Surrey Memorial Hospital during the COVID-19 pandemic between March 1, 2020, and May 1, 2020. We included all COVID-19-positive patients who underwent at least one prone position session within 24 hours of liberation from mechanical ventilation. COVID-19 positivity was defined as a positive real-time reverse transcriptase polymerase chain reaction assay performed on a nasopharyngeal swab or an endotracheal tube aspirate obtained on admission.

We characterized PEPP sessions in terms of duration and number and report pre- and postpositioning ratio of measured oxygen saturation/fraction of inspired oxygen (SpO_2_/FiO_2_), heart rate (HR), and respiratory rate (RR). The values for FiO_2_, HR, and RR were obtained from the critical care flow sheet. We assessed the rate of reintubation within 48 hours and 7 days as compared to COVID-19 patients who were intubated longer than 14 days and did not undergo PEPP in the 24 hours following extubation in the previously published cohort [1].

We performed a qualitative description of the allied health experience with PEPP by scrutinizing the nurse (RN), respiratory therapy (RT), and physiotherapy (PT) notes and rated each comment as being positive, negative, or neutral. Adverse events were defined as any unfavourable or unintended signs, symptoms, or comments from the patients or allied health staff as charted in the record during and for the 12 hours after PEPP.

Numerical data were reported as means and standard deviation (SD) or medians with interquartile range (IQR), as appropriate. Due to the small sample size, effect size was not reported. Instead, individual responses in physiological parameters are presented.

## 3. Results

The literature search, outlined in Figure 1, revealed 1303 titles. 139 abstracts and 43 full-text articles were reviewed. We did not find prior reports of PEPP following prolonged mechanical ventilation.

A total of 4 patients (3 men and 1 woman) underwent prone positioning after liberation from mechanical ventilation. Baseline characteristics and outcome data are reported in Table 1. Included patients underwent mechanical ventilation for a median duration of 25 days (IQR: 22.2, 28) prior to liberation. A total of 2 patients received steroids and 3 underwent prone positioning during mechanical ventilation. All 4 received paralytics for a median duration of 5.5 days (IQR: 1.75, 9.25).

**Figure 2:**
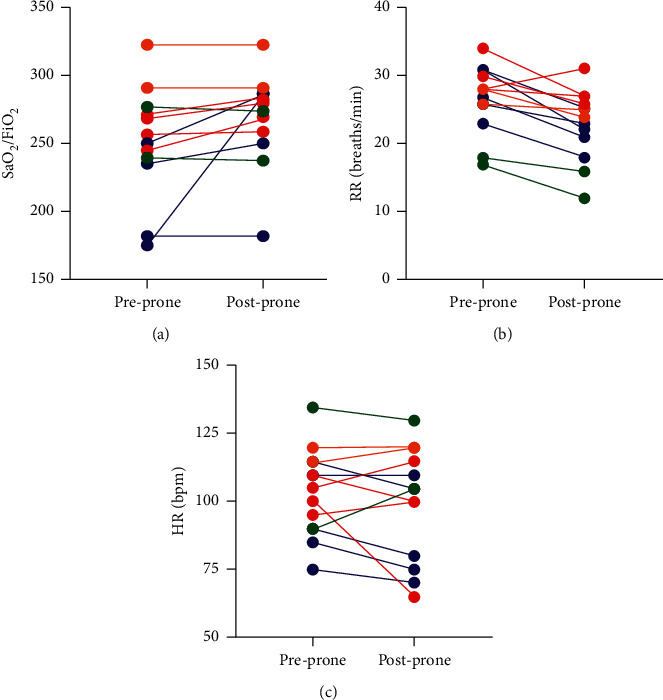
Individual patient values for SaO_2_/FiO_2_, RR, and HR prior to and following each prone positioning session. Each color represents a different patient: blue = patient 1, green = 2, red = 3, and orange = 4.

**Figure 3:**
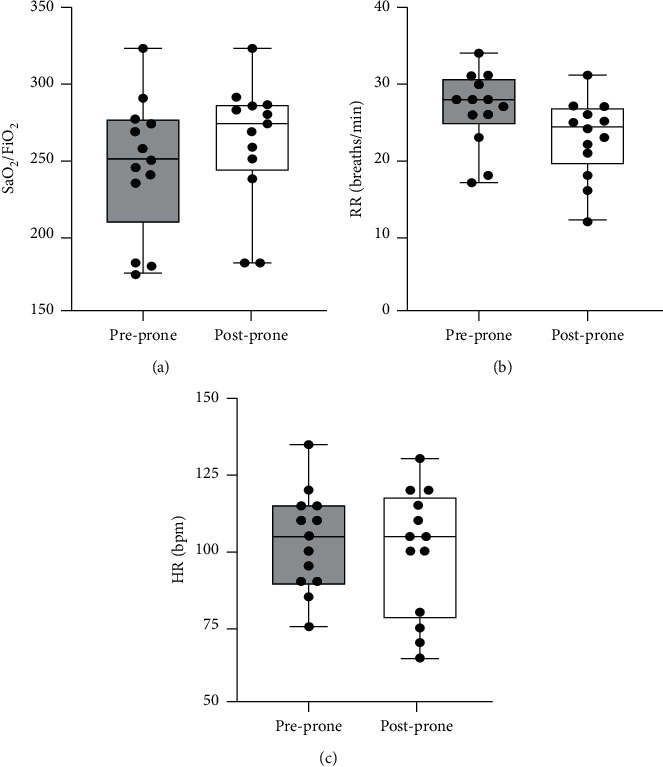
Aggregate patient values for SaO_2_/FiO_2_, RR, and HR prior to and following each prone positioning session.

Each patient underwent a median (IQR) of 3 prone sessions (2, 4.25) lasting 90 minutes (75, 120), over 2.5 days (2, 3.25). The median (IQR) values for FiO_2_, SpO_2_/FiO_2_, HR, and RR were 40% (35, 40), 250 (235, 274), 105 bpm (90, 115), and 28 breaths/minute (26, 30) pre-PEPP and 35% (35, 40), 274 (250, 286), 105 bpm (80, 115), and 24 breaths/minute (21, 26) post-PEPP, respectively. The individual and aggregate patient physiologic parameters associated with PEPP sessions are illustrated in Figures 2 and 3, respectively.

At the time of publication, all patients had been discharged from the ICU with a median (IQR) ICU LOS of 43 days (35.3, 57.8). The fourth patient was reintubated for decreased level of consciousness 9 days after extubation. None of the 4 patients who underwent PEPP following liberation from mechanical ventilation were reintubated within 7 days. This is in comparison with a 7/35 (20%) 48-hour extubation failure rate in COVID-19 respiratory failure patients who were ventilated for over 14 days and did not receive PEPP.

**Figure 4:**
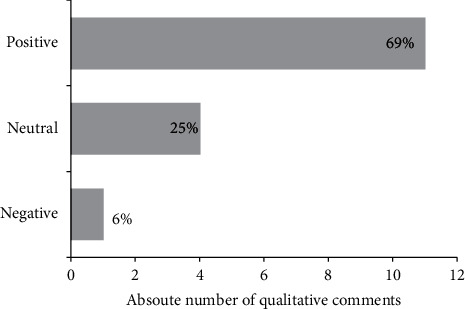
Qualitative rating of allied health personnel comments with respect to postextubation prone positioning abstracted from healthcare records.

None of the patients were able to position themselves in the prone position after extubation due to weakness. Most prone positioning sessions were associated with positive notes by the nursing, physiotherapy, and respiratory therapy staff, and on two occasions, the RN or allied health team extended the prone sessions beyond the duration prescribed by the MD due to perceived benefit to the patient. Qualitative allied healthcare comments are presented in Figure 4. The most commonly reported adverse event was difficulty with neck turning due to stiffness (2 instances), an episode of tachycardia in a patient with known atrial fibrillation (1 instance), and one desaturation event to 86% immediately after return to supine position which was corrected rapidly with suctioning.

## 4. Discussion

Our systematic search of the literature failed to reveal any reports on feasibility, respiratory mechanics, physiologic parameters, or reintubation rates in PEPP for adult patients who have undergone prolonged mechanical ventilation.

In our series, prone positioning was associated with improved SpO_2_/FiO_2_ ratios and HR and reduced RR, as well as reduced reintubation rates. Although our sample size is small, our observations are consistent with data from preintubation prone positioning in COVID-19 patients [10–12]. Other than improved oxygenation, the lower rates of reintubation could potentially be explained by improved secretion clearance and improved pulmonary mechanics during PEPP.

The PEPP sessions in our cohort are shorter than the prone sessions recently reported in COVID-19 patients who were not yet ventilated [9–11]. However, short episodes of prone positioning may be able to result in meaningful physiologic effect. Prior studies demonstrate that clearance of secretions in prone positioning is greatest within the first 2 hours of the session [18], and oxygenation in conscious prone patients improves as quickly as 5 minutes into the session [11]. Additionally, there were minimal reported adverse reactions to the prone sessions, and prone positioning was perceived as beneficial and well tolerated.

A major limitation of our study is the small sample size as well as the relatively short follow-up period. This limits the generalizability and applicability of these data. Due to the early and effective healthcare measures employed in British Columbia, there is currently a paucity of COVID-19 cases in our ICU, limiting further expansion of the current cohort. Due to the retrospective nature of this publication, we were unable to obtain PaO_2_ values before and after each of the PEPP sessions. Instead, we elected to report on SpO_2_/FiO_2_ ratios, which have been shown to correlate with PaO_2_/FiO_2_ ratios [19]. Another limitation and potential confounder is that the benefit seen in clearing secretions and avoiding reintubation could be due to increased patient interaction time by the allied health team during PEPP.

Taken together, this case series suggests that PEPP following liberation from prolonged mechanical ventilation during the COVID-19 pandemic is feasible, well tolerated, and acceptable to the allied healthcare team. Due to the small sample size, conclusions regarding effects on physiologic parameters or reintubation rates cannot be drawn. Larger prospective trials evaluating this novel application of prone positioning are warranted.

## Figures and Tables

**Table 1:** Patient characteristics and descriptive statistics.

	Cohort	Patient 1	Patient 2	Patient 3	Patient 4
Age, median (IQR)	71 (66, 74.25)	57	73	78	69
Sex, female (%)	1 (25)	M	M	M	F
Day 1 APACHE II, median (IQR)	32 (30, 35)	27	31	34	38
Day 1 SOFA II, median (IQR)	11 (11, 11.25)	11	11	12	11
BMI, median (IQR)	22.7 (22.59,25.63)	22.29	22.69	22.79	34.16
Coronary artery disease, *n* (%)	1 (25)	N	N	N	Y
Hypertension, *n* (%)	3 (75)	N	Y	Y	Y
Diabetes, *n* (%)	2 (50)	N	N	Y	Y
Dyslipidemia, *n* (%)	2 (50)	N	Y	Y	N
Obesity, *n* (%)	1 (25)	N	N	N	Y
Duration of MV in days, median (IQR)	25 (22.2, 28)	20	27	31	23^a^
Days in the ICU, median (IQR)	43 (35.3, 57.8)	30	37	49	84
Days of steroid use, median (IQR)	1.5 (0, 4.25)	0	0	8	3
Days with paralytic use, median (IQR)	5.5 (1.75, 9.25)	9	1	10	2
Days undergoing prone positioning while on MV, median (IQR)	4 (2.25, 5)	3	0	5	5
iNO use, *n* (%)	0	N	N	N	N
Hydroxychloroquine use, *n* (%)	0	N	N	N	N
Tocilizumab use, *n* (%)	0	N	N	N	N
PEPP individual sessions, median (range)	3 (2, 4.25)	2	2	4	5
Duration in days of PEPP, median (range)	2.5 (2, 3.25)	2	2	3	4
Duration of individual PEPP sessions in hours, median (IQR)	1.5 (1.2, 2.1)	1.33, 0.42	1, 0.5	2, 1.5, 1.25, 1.5	2.33, 4.08, 3.67, 1.75

IQR = interquartile range, APACHE II = Acute Physiologic and Chronic Health Evaluation II score, SOFA = Sequential Organ Failure Assessment score, BMI = body mass index, MV = mechanical ventilation, ICU = intensive care unit, iNO = inhaled nitric oxide, and PEPP = postextubation prone positioning. ^a^The number of MV days prior to extubation.

## Data Availability

The deidentified patient level data used to support the findings of this study are available from the corresponding author upon request.

## Appendix

### Database Search Terms Used

  EBASE
(1) Humans/
(2) Prone position or prone position.mp.
(3) Mechanical ventilation.mp. or artificial ventilation/
(4) Endotracheal intubation/
(5) Reintubation.mp.
(6) Ventilator weaning.mp. or ventilator weaning/
(7) Extubation.mp. or extubation/
(8) Postextubation.mp.
(9) Following extubation.mp.
(10) 2 or 3
(11) 4 or 5 or 6 or 7 or 8 or 9 or 10
(12) 1 and 11 and 12
  MEDLINE
(1) Exp Humans/
(2) Exp Prone Position/
(3) Ventilator Weaning/
(4) Airway Extubation/
(5) Intubation, Intratracheal/
(6) Prone position.mp.
(7) Postextubation.mp.
(8) Following extubation.mp.
(9) Reintubation.mp.
(10) 2 or 6
(11) 3 or 4 or 5 or 7 or 8 or 9
(12) 1 and 10 and 11
  CENTRAL
(1) Prone position.mp. or Prone Position/
(2) Intubation.mp. or Intubation, Intratracheal/
(3) Extubation.mp. or Airway Extubation/or Respiration, Artificial/
(4) Ventilator weaning.mp. or Ventilator Weaning/
(5) Reintubation.mp.
(6) Postextubation.mp.
(7) Following extubation.mp.
(8) 2 or 3 or 4 or 5 or 6 or 7
(9) 1 and 8
(10) Adult
(11) 9 and 10

## References

[1] Mitra A. R., Fergusson N. A., Lloyd-Smith E. (2020). Baseline characteristics and outcomes of patients infected with SARS-CoV2 admitted to intensive care units in Metro Vancouver, Canada: a case series. *The Canadian Medical Association Journal*.

[2] Kallet R. H. (2015). A comprehensive review of prone position in ARDS. *Respiratory Care*.

[3] Gattinoni L., Busana M., Giosa L., Macrì M., Quintel M. (2019). Prone positioning in acute respiratory distress syndrome. *Seminars in Respiratory and Critical Care Medicine*.

[4] Koulouras V., Papathanakos G., Papathanasiou A., Nakos G. (2016). Efficacy of prone position in acute respiratory distress syndrome patients: a pathophysiology-based review. *World Journal of Critical Care Medicine*.

[5] Scholten E. L., Beitler J. R., Prisk G. K., Malhotra A. (2017). Treatment of ARDS with prone positioning. *Chest*.

[6] Albert R. K., Hubmayr R. D. (2000). The prone position eliminates compression of the lungs by the heart. *American Journal of Respiratory and Critical Care Medicine*.

[7] Cornejo R. A., Díaz J. C., Tobar E. A. (2013). Effects of prone positioning on lung protection in patients with acute respiratory distress syndrome. *American Journal of Respiratory and Critical Care Medicine*.

[8] Jozwiak M., Teboul J.-L., Anguel N. (2013). Beneficial hemodynamic effects of prone positioning in patients with acute respiratory distress syndrome. *American Journal of Respiratory and Critical Care Medicine*.

[9] Sun Q., Qiu H., Huang M., Yang Y. (2020). Lower mortality of COVID-19 by early recognition and intervention: experience from Jiangsu Province. *Annals of Intensive Care*.

[10] Sartini C., Tresoldi M., Scarpellini P. (2020). Respiratory parameters in patients with COVID-19 after using noninvasive ventilation in the prone position outside the intensive care unit. *JAMA*.

[11] Caputo N. D., Strayer R. J., Levitan R. (2020). Early self-proning in awake, non-intubated patients in the emergency department: a single ED’s experience during the COVID-19 pandemic. *Academic Emergency Medicine*.

[12] Paul V., Patel S., Royse M., Odish M., Malhotra A., Koenig S. (2020). Proning in non-intubated (PINI) in times of COVID-19: case series and a review. *Journal of Intensive Care Medicine*.

[13] Van Der Burg P. S., Miedema M., De Jongh F. H., Frerichs I., Van Kaam A. H. (2015). Changes in lung volume and ventilation following transition from invasive to noninvasive respiratory support and prone positioning in preterm infants. *Pediatric Research*.

[14] Feltracco P., Serra E., Barbieri S. (2012). Noninvasive high-frequency percussive ventilation in the prone position after lung transplantation. *Transplantation Proceedings*.

[15] Wang D., Hu B., Hu C. (2020). Clinical characteristics of 138 hospitalized patients with 2019 novel coronavirus-infected pneumonia in Wuhan, China. *Jama*.

[16] Hermans G., Van den Berghe G. (2015). Clinical review: intensive care unit acquired weakness. *Critical Care*.

[17] Thille A. W., Boissier F., Muller M. (2020). Role of ICU-acquired weakness on extubation outcome among patients at high risk of reintubation. *Critical Care*.

[18] Gillart T., Bazin J. E., Guelon D. (2000). Influence du drainage bronchique dans l'amélioration des échanges gazeux observée en décubitus ventral au cours du SDRA. *Annales Françaises d’Anesthésie et de Réanimation*.

[19] Brown S. M., Grissom C. K., Moss M. (2016). Nonlinear imputation of PaO2/FiO2 from SPO2/FiO2 among patients with acute respiratory distress syndrome. *Chest*.

